# Efficacy and safety of perampanel as early add-on therapy in Chinese patients with focal-onset seizures: a multicenter, open-label, single-arm study

**DOI:** 10.3389/fneur.2023.1236046

**Published:** 2023-08-30

**Authors:** Lehong Gao, Qiang Lu, Zan Wang, Wei Yue, Guoping Wang, Xiaoqiu Shao, Yi Guo, Yonghong Yi, Zhen Hong, Yuwu Jiang, Bo Xiao, Guiyun Cui, Feng Gao, Jiasheng Hu, Jianmin Liang, Meiyun Zhang, Yuping Wang

**Affiliations:** ^1^Department of Neurology, Xuanwu Hospital, Capital Medical University, Beijing, China; ^2^Department of Neurology, Peking Union Medical College Hospital, Beijing, China; ^3^Department of Neurology, The First Hospital of Jilin University, Changchun, China; ^4^Department of Neurology, Tianjin Huanhu Hospital, Tianjin, China; ^5^Division of Life Sciences and Medicine, Department of Neurology, The First Affiliated Hospital of USTC, University of Science and Technology of China, Hefei, China; ^6^Department of Neurology, Beijing Tiantan Hospital, Capital Medical University, Beijing, China; ^7^Department of Neurology, Shenzhen People's Hospital, The Second Clinical Medical College, Jinan University, The First Affiliated Hospital, Southern University of Science and Technology, Shenzhen, China; ^8^Key Laboratory of Neurogenetics and Channelopathies of Guangdong Province and the Ministry of Education of China, Department of Neurology, Institute of Neuroscience, The Second Affiliated Hospital of Guangzhou Medical University, Guangzhou, China; ^9^Department of Neurology, Huashan Hospital, Fudan University, Shanghai, China; ^10^Department of Pediatrics and Pediatric Epilepsy Center, Peking University First Hospital, Beijing, China; ^11^Department of Neurology, Xiangya Hospital, Central South University, Changsha, China; ^12^Department of Neurology, The Affiliated Hospital of Xuzhou Medical University, Xuzhou, China; ^13^Department of Neurology, Children's Hospital, Zhejiang University School of Medicine, Hangzhou, China; ^14^Department of Neurology, Wuhan Children's Hospital, Tongji Medical College, Huazhong University of Science and Technology, Wuhan, China; ^15^Department of Pediatric Neurology, The First Hospital of Jilin University, Changchun, China; ^16^Department of Neurology, Tianjin Union Medical Center, Tianjin, China; ^17^Beijing Key Laboratory of Neuromodulation, Beijing, China; ^18^Center of Epilepsy, Institute of Sleep and Consciousness Disorders, Beijing Institute for Brain Disorders, Capital Medical University, Beijing, China; ^19^Neuromedical Technology Innovation Center of Hebei Province, Hebei Hospital of Xuanwu Hospital, Capital Medical University, Shijiazhuang, China

**Keywords:** perampanel, focal-onset seizures, Chinese patients, perampanel early add-on, 50% responder rate, seizure-freedom rate perampanel, seizure-freedom rate

## Abstract

**Background:**

No interventional study has been conducted in China to assess efficacy and safety of perampanel in treating Chinese patients with epilepsy, nor has there been any study on perampanel early add-on therapy in China. This interventional study aimed to assess efficacy and safety of perampanel as an early add-on treatment of focal-onset seizures (FOS) with or without focal-to-bilateral tonic–clonic seizures (FBTCS) in Chinese patients.

**Methods:**

In this multicenter, open-label, single-arm, phase 4 interventional study, Chinese patients ≥ 12 years old with FOS with or without FBTCS who failed anti-seizure medication (ASM) monotherapy from 15 hospitals in China were enrolled and treated with perampanel add-on therapy (8-week titration followed by 24-week maintenance). The primary endpoint was 50% responder rate. Secondary endpoints included seizure-freedom rate and changes in seizure frequency from baseline. Treatment-emergent adverse events (TEAEs) and drug-related TEAEs were recorded.

**Results:**

The full analysis set included 150 patients. The mean maintenance perampanel dose was 5.9 ± 1.5 mg/day and the 8-month retention rate was 72%. The 50% responder rate and seizure-freedom rate for all patients during maintenance were 67.9 and 30.5%, respectively. Patients with FBTCS had higher 50% responder rate (96.0%) and seizure-freedom rate (76.0%) during maintenance. Patients on concomitant sodium valproate had a significantly higher seizure-freedom rate than those on concomitant oxcarbazepine. Eight-six (55.1%) patients experienced treatment-related TEAEs, and the most common TEAEs were dizziness (36.5%), hypersomnia (11.5%), headache (3.9%), somnolence (3.2%), and irritability (3.2%). Withdrawal due to TEAEs occurred to 14.7% of the patients.

**Conclusion:**

Perampanel early add-on was effective and safe in treating Chinese patients≥12 years old with FOS with or without FBTCS.

**Clinical trial registration**www.chictr.org.cn, Identifier ChiCTR2000039510.

## 1. Introduction

Epilepsy is a common chronic brain disease affecting more than 50 million people worldwide (1). It is characterized by recurrent, sudden unprovoked seizures (1). There are approximately 6 million patients with active epilepsy (≥2 unprovoked seizures in the previous year) in China, 60% of whom have focal-onset seizures (FOS) (2). Patients with epilepsy are more likely to suffer additional physical and psychological problems and they are 2–3 times more likely to suffer premature death compared to general population (1, 2). The primary treatment for epilepsy is anti-seizure medications (ASMs), and ASM monotherapy is the gold standard for treating patients with newly diagnosed epilepsy (3). Around 50%–60% of patients with epilepsy achieved sustained seizure-freedom on their first ASM monotherapy (4, 5). When the first ASM failed, dose increase, a different ASM or combination treatment using ≥2 ASMs with distinct mechanisms of action could be tried (6–8). Traditionally, combination therapy is often used when 2–3 ASM monotherapy regimens failed to control seizures in a patient (7). However, some have argued for earlier use of combination therapy especially in patients with severe epilepsy who could tolerate their first ASM and were partially responsive (6, 7). It has been reported that combination therapy was more likely to help patients who failed initial ASM monotherapy due to lack of efficacy to achieve seizure freedom than alternative monotherapy or initial ASM at larger dose (8). This is probably because newer ASMs with distinct mechanisms of action could increase chance of seizure freedom in patients receiving combination therapies compared to traditional ASMs (9).

Perampanel is a first-in-class, orally active, highly selective, noncompetitive antagonist of alpha-amino-3-hydroxy-5-methyl-4-isoxazolepropionic acid (AMPA)-type glutamate receptor (10, 11). AMPA receptors are the main excitatory post-synaptic glutamate receptors that mediate the fast excitatory synaptic transmission and play important roles in triggering and spreading epileptic seizures (10–12). Perampanel inhibits excitatory neurotransmission by targeting post-synaptic glutamate activity, additionally, it could block increased level of glutamate because it is non-competitive and could not be displaced under high concentration of AMPA receptor agonist, and therefore it possesses strong anti-seizure activities (12). Numerous preclinical animal models, phase 3 clinical studies and observational studies demonstrated that perampanel had broad-spectrum anti-seizure activities in animal models and patients with focal and generalized seizures (3, 10, 12).

Perampanel, as a once daily oral ASM is indicated for monotherapy and adjunctive treatment of FOS with or without focal-to-bilateral tonic–clonic seizures (FBTCS) in patients aged 4 years or older in China (13, 14). Many studies including several phase III randomized controlled studies found that perampanel as monotherapy or add-on treatment at 4–12 mg/day was effective and safe in treating FOS with or without FBTCS (10–12, 14–19). Long-term seizure control by adjunctive perampanel therapy and its safety have also been demonstrated (20–22). Fernandes et al. reported that perampanel in association with 1 or 2 ASMs showed good efficacy and safety in pediatric and adult patients through a follow-up period of 24 months or longer in a real-world observational study (20). The extension phase of study 235, a multicenter, randomized, double-blind Phase II study demonstrated good seizure-control at week 40–52 associated with adjunctive perampanel therapy in adolescent patients with FOS (22). Additionally, as it is well known that ASMs could be detrimental to cognitive function, especially in children and adolescents whose brains are still developing (22, 23), and data on safety cannot be extrapolated from adult to pediatric patients (24), it is important to evaluate safety of adjunctive perampanel in pediatric patients. Studies have reported that adjunctive perampanel therapy did not adversely affect executive functions in adolescents with resistant FOS during a 12-month treatment period (25) and that perampanel first add-on was effective in treating children with absence seizure and had a good tolerability profile, that is the treatment did not adversely affect the patients’ “non-verbal intelligence, executive functions, emotional/behavioral symptoms of children and parental stress levels” (26). Therefore, adjunctive perampanel therapy has been demonstrated as an effective and safe treatment for both adult and pediatric patients with FOS. However, in China, as perampanel was not approved for treating FOS until September 2019 (13), there have been only 4 observational studies on the efficacy and safety of adjunctive perampanel in treating FOS in Chinese adult patients (11, 14, 18, 19) and 6 observational studies on perampanel add-on in treating Chinese pediatric patients with epilepsy (27–32), none of which was on perampanel early add-on therapy. In the current multicenter, open-label, single-arm interventional study, we aimed to assess efficacy and safety of perampanel as an early add-on treatment for FOS with or without FBTCS in Chinese adolescent and adult patients. Such a study could help clinicians in China to better understand application of perampanel in treating Chinese patients with FOS.

## 2. Materials and methods

### 2.1. Study design, patients and treatment

**Figure 1:**
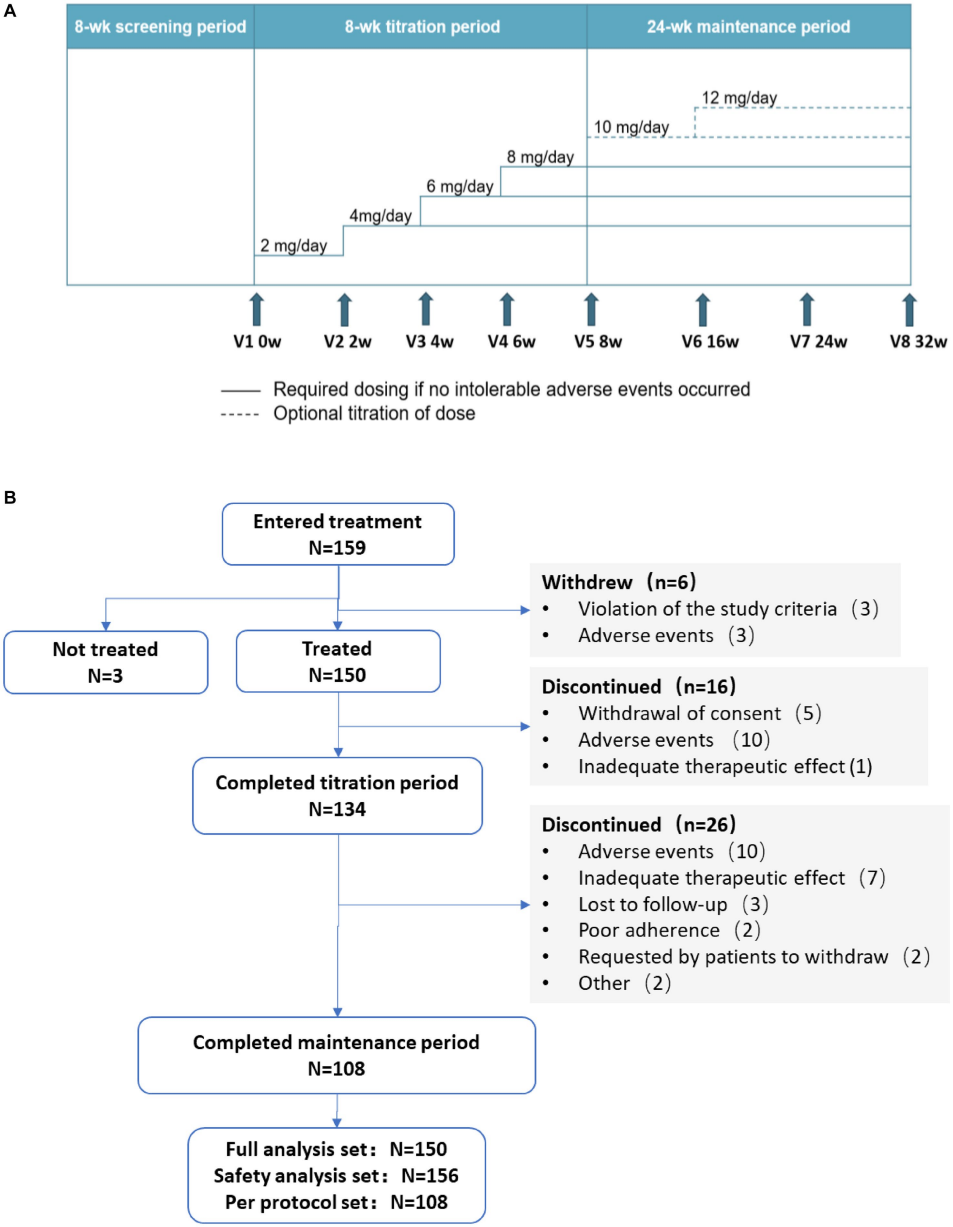
**(A)** Study design and **(B)** study flow chart. wk., week; V, visit; w, week.

This is a multicenter, open-label, single-arm, prospective, phase 4 clinical trial conducted from June 2020 to March 2022. The first patient in the study was enrolled on 16 December 2020 and follow-ups for the last patient were completed on 29 November 2022. The study consisted of an 8-week screening period (the 8 weeks before 0 week) followed by two treatment periods: an 8-week titration period (0 week to 8 weeks) and a 24-week maintenance period (8 to 32 weeks; Figure 1A).

This study enrolled patients who visited 15 tertiary hospitals in 12 provinces in China (participating hospitals are listed in Acknowledgements). Inclusion criteria: (1) Patients≥12 years old diagnosed with FOS with or without FBTCS according to the 2017 International League Against Epilepsy (ILAE) classification of seizure types (33) who failed to achieve seizure control with ASM monotherapy and needed add-on therapy; (2) patients must have been receiving ASD monotherapy at a stable dose for at least 4 weeks prior to Week 0 and ≤2 ASM monotherapy regimens were allowed during those 4 weeks and no other additional anti-seizure treatment was allowed; and (3) patients must have experienced an average of ≥2 FOS per month during the screening period and the interval between the 2 FOS should be >24 h.

Exclusion criteria: (1) Pregnant (β-human chorionic gonadotropin positive) or lactating female patients; (2) patients with idiopathic generalized epilepsy (IGE) such as absence and/or myoclonic epilepsy; (3) patients with a history of status epilepticus within 12 weeks prior to Week 0; (4) patients who took antipsychotic medication(s), who were diagnosis with a mental disorder, or who were diagnosed with unstable recurrent affective disorder(s) with a history of suicide attempt(s) within 1 year before Week 0; (5) patients with progressive central nervous system (CNS) disease(s) including CNS degenerative disease(s) and progressing tumor(s); (6) patients who took concomitant barbiturate(s) or benzodiazepines except for the purpose of treating convulsion or electroencephalogram (EEG) during the screening period; (7) patients who were administered with benzodiazepines during emergency treatment for at least 2 times during the screening period; (8) patients with moderate to severe renal diseases or patients who had been receiving hemodialysis; (9) patients with serious liver disease(s); (10) patients with lactose intolerance, lactose deficiency or glucose-galactose malabsorption; (11) patients who were allergic to ingredient(s) of perampanel; (12) patients who were participating in other clinical trials; and (13) patients deemed unsuitable for the current study as judged by our investigators.

Study termination criteria: (1) withdrawal of informed consent by patients who requested to withdraw from the study; (2) withdrawal due to adverse events out of safety concerns as judged by our investigators; (3) poor adherence to dosing (<80% or >120%); (4) use of medication(s) that might affect efficacy evaluation of perampanel; (5) lost to follow-up; (6) pregnancy; and (7) patients could not tolerate 4 mg/day perampanel.

During the screening period, patients continued their existing ASM monotherapy, and their demographics and baseline characteristics including epilepsy-related information and previous ASM regimen(s) were collected.

During titration and maintenance, all patients took oral perampanel once daily at bedtime. During titration, with a starting dose of 2 mg/day, dose of perampanel was increased in 2 mg increments at intervals of ≥2 weeks based on patient’s tolerance until a maximum dose of 8 mg/day was reached. Patients who experienced intolerable adverse events (AEs) could either maintain their current dose without further up-titration or have their daily dose of perampanel reduced to a previous dose that could be tolerated. Patients who could not tolerate 4 mg/day perampanel were excluded from the study.

Patients entered the 24-week maintenance period on the maximum dose achieved during the titration period. Patients experiencing intolerable AEs could consider reducing their dosage. For patients whose seizures were not completely controlled, perampanel dose could be further up-titrated in 2 mg increments at intervals of ≥2 weeks, to a maximum maintenance dose of 12 mg/day. The dosage of the concomitant ASM(s) each patient received during this period was fixed and could not be adjusted. No other anti-seizure treatment was allowed during the study.

Perampanel was considered as first add-on in patients who had previously received only 1 ASM monotherapy regimen and perampanel was the second ASM for these patients, and perampanel was considered as second add-on in patients who had received 2 ASM monotherapy regimens and perampanel was the third ASM for these patients.

This study was reviewed and approved by the Institutional Review Boards for Ethical Approval of all participating hospitals in 2020. The participating hospitals were as follows: (1) Xuanwu Hospital, Capital Medical University, (2) Peking Union Medical College Hospital, (3) The First Hospital Of Jilin University, (4) Tianjin Huanhu Hospital, (5) The First Affiliated Hospital of USTC, Division of Life Sciences and Medicine, University of Science and Technology of China, (6) Beijing Tiantan Hospital, Capital Medical University, (7) Shenzhen People’s Hospital, The Second Clinical Medical College, Jinan University, The First Affiliated Hospital, Southern University of Science and Technology, (8) The Second Affiliated Hospital of Guangzhou Medical University, Key Laboratory of Neurogenetics and Channelopathies of Guangdong Province and the Ministry of Education of China, (9) Huashan Hospital, Fudan University, (10) Peking University First Hospital, (11) Xiangya Hospital, Central South University, (12) The Affiliated Hospital of Xuzhou Medical University, (13) Children’s Hospital, Zhejiang University School of Medicine, (14) Wuhan Children’s Hospital, Tongji Medical College, Huazhong University of Science and Technology, and (15) Tianjin Union Medical Center.

The study was conducted in accordance with the principles of the Declaration of Helsinki. All patients or their legal guardian/next of kin gave written informed consent to participate in this study before enrollment. This trial was registered at Chinese Clinical Trial Registry (www.chictr.org.cn, Identifier: ChiCTR2000039510).

### 2.2. Efficacy endpoints

The primary endpoint was 50% responder rate (the proportion of patients who had ≥50% reduction of seizure frequency from baseline during the maintenance period).

The secondary endpoints consisted of: (1) seizure-freedom rate (the proportion of patients who remained seizure-free during the maintenance period); (2) changes in seizure frequency from baseline at the end of titration period and at the end of the maintenance period, calculated using the following formula: ([seizure frequency during the titration/maintenance period] – [baseline seizure frequency])/(baseline seizure frequency) × 100%; (3) 50% response rate and seizure-free rate of patients with FBTCS, and changes in seizure frequency from baseline at the end of titration period and at the end of the maintenance period for patients with FBTCS; (4) 8-month retention rates [the proportion of patients who remained on perampanel add-on therapy after 8 months of treatment (titration period + maintenance period)].

Seizure frequency during the titration/maintenance period was expressed as number of seizures every 28 days within that period using the following formula: seizure frequency = (total number of seizures during titration/maintenance)/(number of days in the period) × 28.

Efficacy subgroup analyses based on patients’ age (patients who were 12–18 years old vs. those who were >18 years old), whether perampanel was first add-on or second add-on, and concomitant ASMs were also performed.

### 2.3. Safety

Safety of the treatment was assessed by monitoring vital signs (blood pressure and heart rate), laboratory tests (blood tests and blood biochemistry), electrocardiograph and documentation of any AEs.

Safety endpoints included incidence of treatment emergent AEs (TEAEs), treatment-related TEAEs, and percentage of patients who withdrew from the study due to TEAEs.

### 2.4. Statistical analyses

Sample size needed for the primary endpoint (50% responder rate) in this study was calculated based on a pooled analysis of three phase 3 studies that analyzed efficacy and safety of perampanel add-on therapy for treating patients with refractory FOS (15, 34). It found that 50% responder rates of patients receiving 8 mg/day perampanel and patients receiving placebo were 35.3% and 19.3%, respectively (15, 34). If the lower limit of the 95% confidential interval (CI) of the 50% responder rate in our study was to be >19.3%, a sample size of ≥94 patients are needed to achieve a statistical power of 90%. Anticipating a dropout rate of 30%, a minimum of 122 patients were needed.

Efficacy analyses were performed using full analysis set (FAS) consisting of patients who received at least one dose of perampanel and who had at least one efficacy assessment, while safety analyses were conducted using safety analysis set (SAS) consisting of patients who received at least one dose of perampanel and who had at least one post-dosing safety assessment.

All statistical analyses in the study were performed using SAS 9.4 (SAS Institute, Cary, NC, United States). Descriptive analysis was used. Categorical variables were expressed as *N* (%), while quantitative variables were expressed as mean ± standard deviation (SD) or median [interquartile range (IQR)]. Time to event was assessed by Kaplan–Meier curves. Between-group comparisons were performed using Chi-square test or Fisher’s exact test for categorical variables and Student t or Mann–Whitney *U*-test for continuous variables. The Bonferroni test was used for multiplicity correction. Retention rate was assessed with the Kaplan–Meier method. Missing data were not imputed, and all analyses were conducted using available data. All statistical analyses were conducted against a two-sided alternative hypothesis with a *p*-value < 0.05 considered to be statistically significant.

## 3. Results

### 3.1. Patients

Study design and flow chart were depicted in Figures 1A,B, respectively. A total of 159 patients were enrolled in the study, among them, 3 patients did not receive any perampanel treatment. Therefore, the SAS included 156 patients. Six patients were excluded as 3 violated inclusion criteria after their enrollment and 3 withdrew due to AEs, so the FAS included the remaining 150 patients. Among these 150 patients, 16 and 26 patients withdrew from the study during titration and maintenance, respectively. The remaining 108 patients completed the study and they constituted the per protocol set (PPS). During the treatment phase, the most common reasons for withdrawal were AEs (*n* = 20), inadequate therapeutic effect (*n* = 8), withdrawal of consent (*n =* 5) and lost to follow-up (*n* = 3; Figure 1B).

Patient demographics and baseline characteristics were described in Table 1. The FAS included 150 patients (74 female and 76 male). Among them, 26 (17.3%) patients were adolescents (12–18 years old) and 124 (82.7%) patients were adults. They had a mean age of 34.7 ± 15.9 years old and a medium seizure frequency of 3.0 (IQR 2.0–9.5) per 28 days. 41 (21.8%) patients experienced focal aware seizures, 100 (68.5%) experienced focal impaired awareness seizures (FIAS), and 27 (18.5%) patients experienced FBTCS. Perampanel was first add-on in 136 (90.7%) patients. The most commonly used concomitant ASMs were levetiracetam (29.3%), oxcarbazepine (29.3%), and sodium valproate (20.7%; Table 1). In addition, baseline characteristics of patients in the “≥18 years old” and “<18 years old” subgroups were also listed in Table 1.

**Table 1:** Demographic and baseline characteristics (full analysis set).

Characteristics	Total (*N* = 150)	≥18 years old (*N* = 124)	<18 years old (*N* = 26)
Age (years), mean ± SD	34.7 ± 15.9	38.96 ± 14.11	14.27 ± 1.71
**Sex, *n* (%)**			
Female	74 (49.3%)	64 (51.6%)	10 (38.5%)
Male	76 (50.7%)	60 (48.4%)	16 (61.5%)
Seizure frequency per 28 days, medium (IQR)	3 (2.0, 9.5)	3 (2.0,7.8)	6.8 (2.5, 11.0)
**ILAE classification, *n* (%)**			
Focal aware seizures	41 (21.8%)	30 (25.0%)	11 (42.3%)
FIAS	100 (68.5%)	85 (70.8%)	15 (57.7%)
FBTCS	27 (18.5%)	19 (15.8%)	8 (30.8%)
**Number of concomitant ASMs, *n* (%)**			
1	136 (90.7%)	113 (91.1%)	23 (88.5%)
≥2	14 (9.3%)	11 (8.9%)	3 (11.5%)
**Baseline concomitant ASMs, *n* (%)**			
Levetiracetam	44 (29.3%)	37 (29.8%)	7 (26.9%)
Oxcarbazepine	44 (29.3%)	33 (26.6%)	11 (42.3%)
Sodium valproate	31 (20.7%)	25 (20.2%)	6 (23.1%)
Lamotrigine	23 (15.3%)	20 (16.1%)	3 (11.5%)
Carbamazepine	17 (11.3%)	17 (13.7%)	0 (0.0%)
Topiramate	4 (2.7%)	4 (3.2%)	0 (0.0%)
Lacosamide	3 (2.0%)	2 (1.6%)	1 (3.9%)
Phenytoin	2 (1.3%)	1 (0.8%)	1 (3.9%)

SD, standard deviation; IQR, interquartile range; ILAE, International League Against Epilepsy; FIAS, focal impaired awareness seizures; FBTCS, focal to bilateral tonic–clonic seizures; ASMs, anti-seizure medications.

### 3.2. Perampanel dose and retention rate

The mean maintenance perampanel dose was 5.9 ± 1.5 mg/day, and 87 (65.4%) patients received ≥6 mg/day perampanel during maintenance.

**Figure 2:**
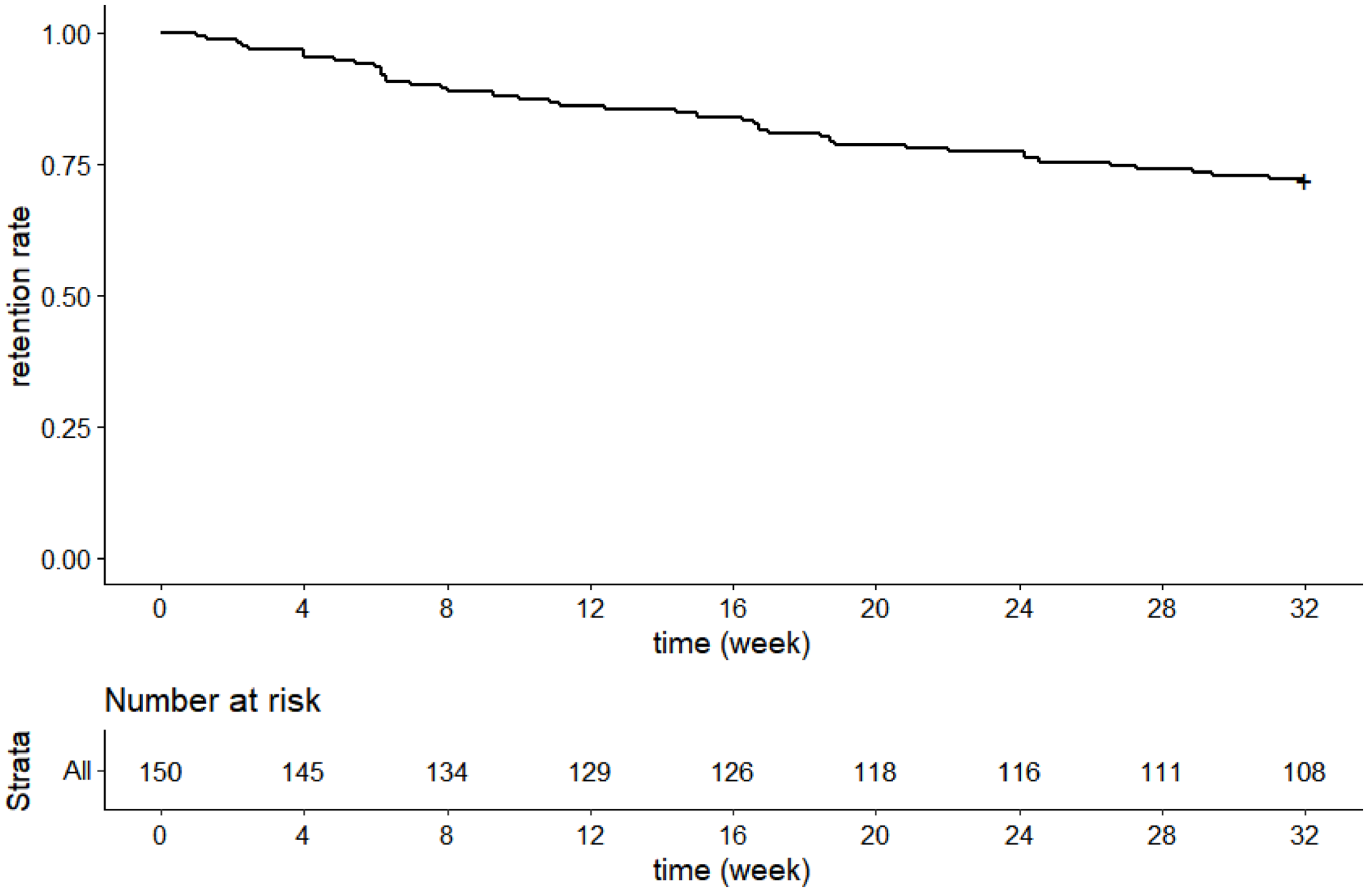
Kaplan–Meier survival curve of perampanel retention (full analysis set).

One hundred and eight patients completed the 8-month treatment, so our study had a 8-month retention rate of 72% (Figure 2).

### 3.3. Efficacy outcomes

**Figure 3:**
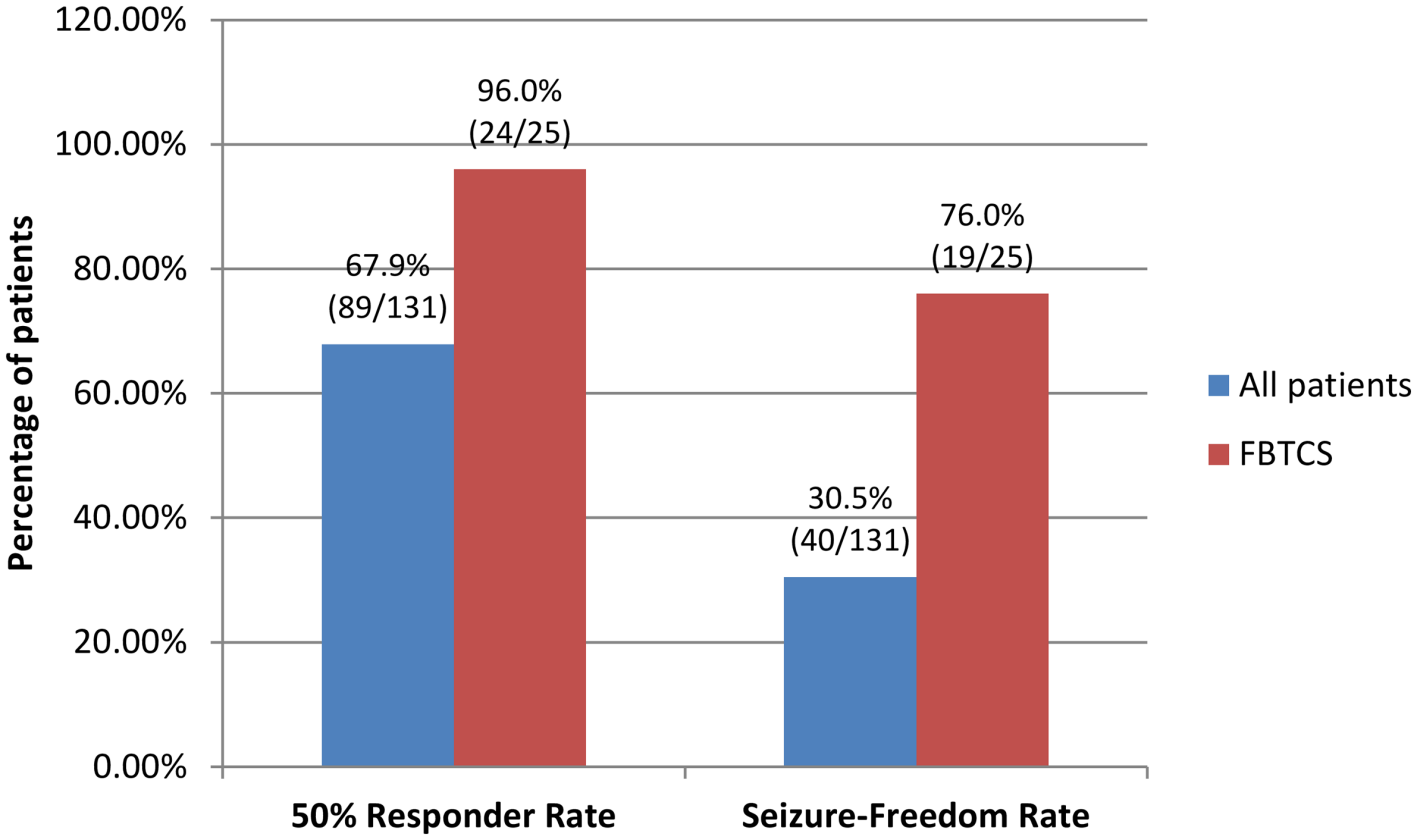
50% Responder rates and seizure-freedom rates of all patients and patients with FBTCS by the end of the 24-week maintenance period. FBTCS, focal to bilateral tonic–clonic seizures.

The 50% responder rate and seizure-freedom rate for all patients in the FAS during maintenance were 67.9% (89/131) and 30.5% (40/131), respectively. Among these patients, patients with FBTCS had higher 50% responder rate and seizure-freedom rate during maintenance, which were 96.0% (24/25) and 76.0% (19/25), respectively (Figure 3).

The FAS population had significantly lower medium seizure frequencies per 28 days during titration (2.0 [IQR 0.5–7.6]) and maintenance (1.2 [IQR 0.0–5.2]) than baseline (3.0 [IQR 2.0–9.5]; both *p* < 0.001). The medium percentage decreases in seizure frequency from baseline during titration and maintenance were 61% (IQR 3%–97%) and 75% (IQR 17%–100%), respectively.

Patients with FBTCS also had a significantly lower medium seizure frequencies per 28 days during titration (0.0 [IQR 0.00–0.5]) and maintenance (0.0 [IQR 0.0–0.0]) than baseline (2.0 [IQR 0.5–3.5]; both *p* < 0.001). The medium percentage decrease from baseline during titration and maintenance were 100% (IQR 85%–100%) and 100% (IQR 100%–100%), respectively.

**Figure 4:**
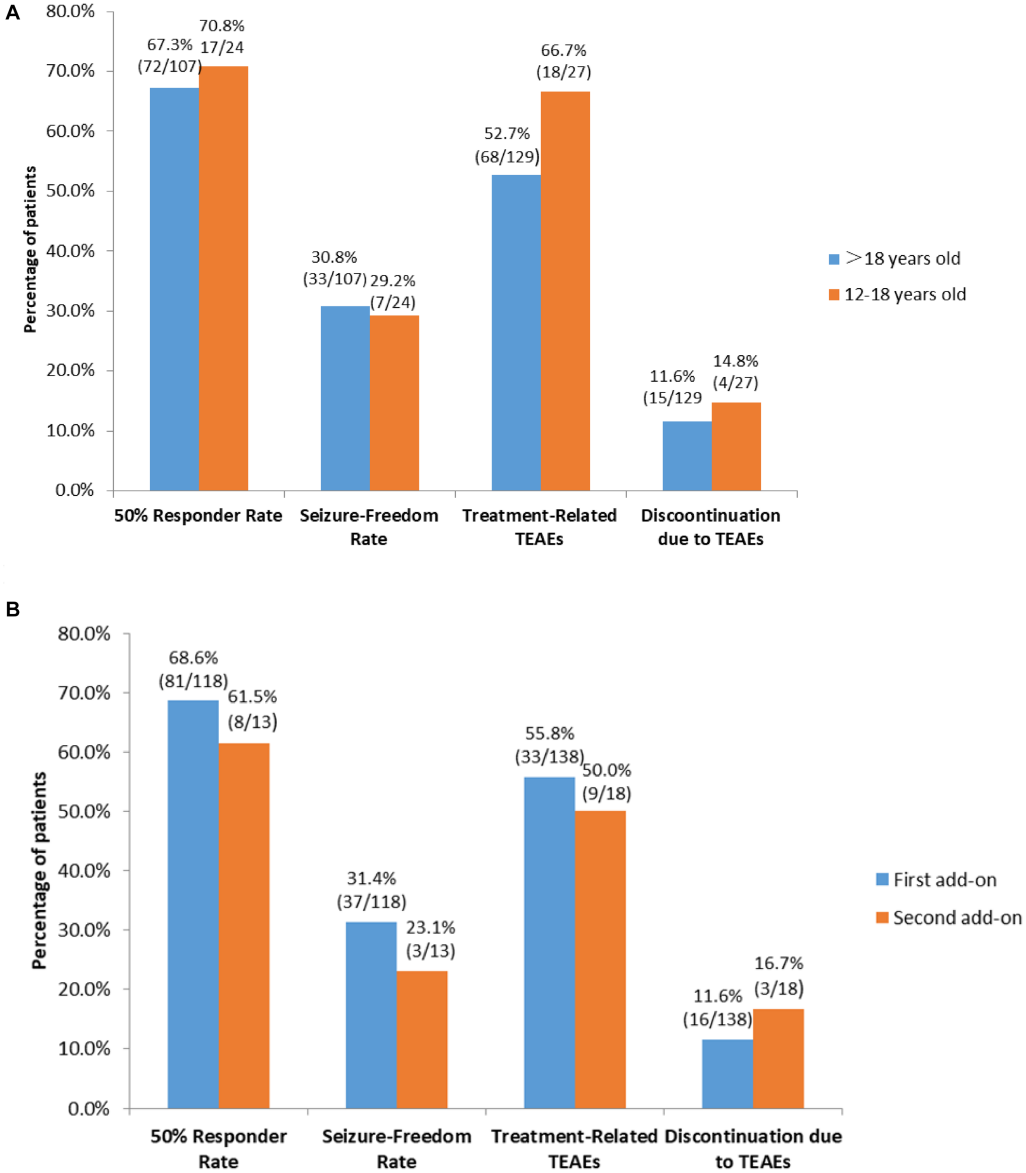
Subgroup analyses of efficacy and tolerability of perampanel early add-on in **(A)** patients > 18 years old vs. patients who were 12–18 years old, and **(B)** patients receiving perampanel as first add-on therapy vs. those receiving perampanel as second add-on therapy. TEATs, treatment emergent adverse events.

Subgroup analyses showed that there were no significant differences in 50% responder rates and seizure-freedom rates between patients who were 12–18 years old and those who were >18 years old (67.3% [72/107] vs. 70.8% [17/24], 30.8% [33/107] vs. 29.2% [7/24], respectively, both *p* > 0.05; Figure 4A), and between patients receiving perampanel as first add-on and those receiving perampanel as second add-on (68.6% [81/118] vs. 61.5% [8/13]. 31.4% [37/118] vs. 23.1% [3/13], respectively, both *p* > 0.05; Figure 4B).

**Figure 5:**
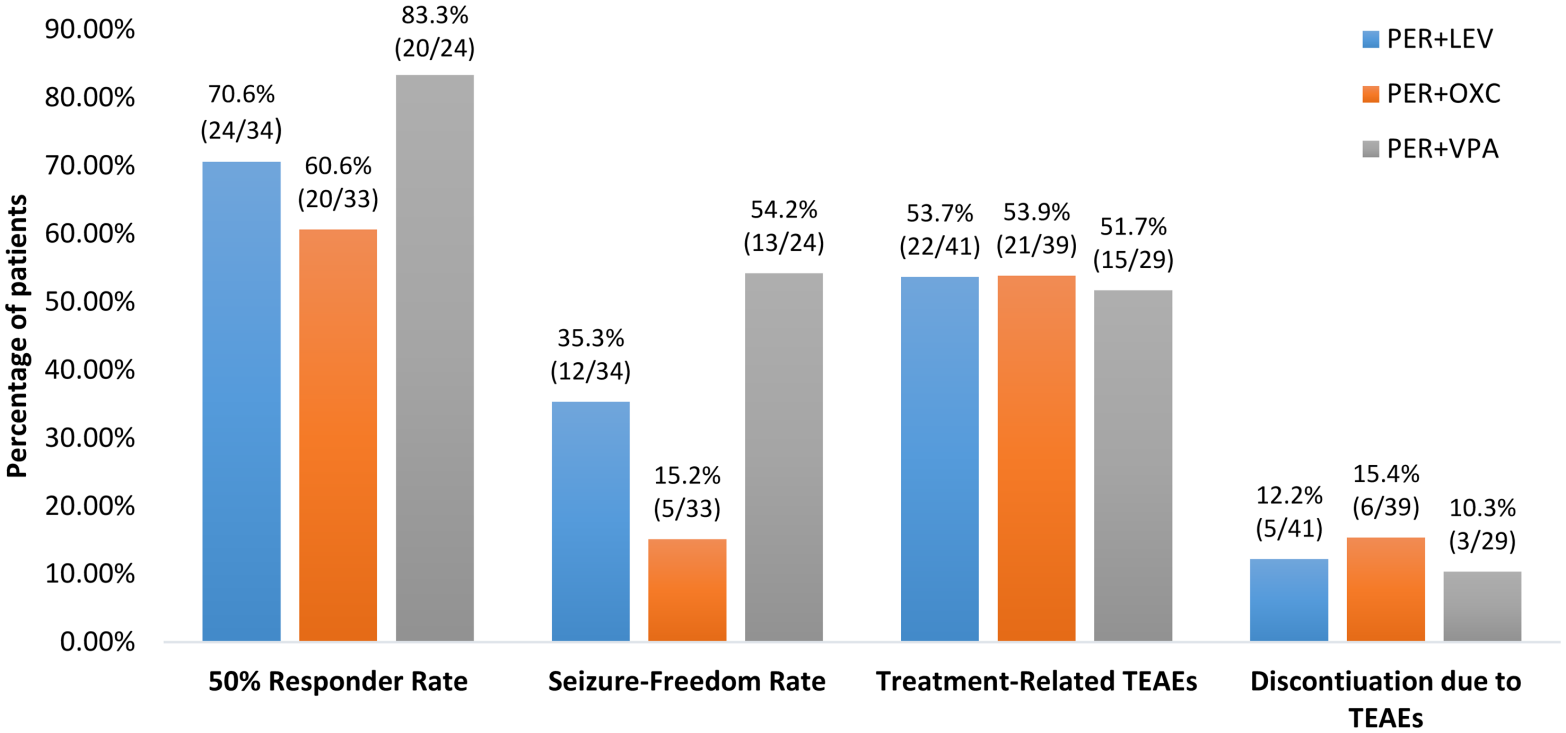
Efficacy and tolerability of perampanel early add-on stratified by different concomitant anti-seizure medications. PER, perampanel; LEV, levetiracetam; OXC, oxcarbazepine; VPA, valproic acid, TEATs, treatment emergent adverse events.

Finally, although there was no significant difference in 50% responder rates among patients on concomitant sodium valproate (83.3% [20/24]), patients on concomitant levetiracetam (70.6% [24/34]) and those on concomitant oxcarbazepine (60.6% [20/33]), patients on concomitant sodium valproate had a significantly higher seizure-freedom rate than those on concomitant oxcarbazepine (54.2% [13/24] vs15.2% [5/33], *p* = 0.02; Figure 5).

### 3.4. Safety

One hundred and nineteen (76.3%) patients experienced TEAEs, among them, 86 (55.1%) patients experienced treatment-related TEAEs, and the most common TEAEs were dizziness (57 [36.5%]), hypersomnia (18 [11.5%]), headache (6 [3.9%]), somnolence (5 [3.2%]), and irritability (5 [3.2%]). Mild TEAEs occurred in 44 (60.3%) patients, and 23 (14.7%) patients withdrew from the study due to TEAEs (Table 2).

**Table 2:** Treatment-emergent adverse events (TEAEs; safety analysis set).

	Total patients (*N* = 156)
TEAE	119 (76.3%)
Treatment-related TEAEs	86 (55.1%)
**Severity of TEAEs**	
Mild	94 (60.3%)
Moderate	40 (25.6%)
Severe	11 (7.1%)
Severe treatment-related TEAEs	2 (1.3%)
TEAEs leading to withdraw from the study	23 (14.7%)
**Most common treatment-related TEAEs (≥2% of patients), *n* (%)**	
Dizziness	57 (36.5%)
Hypersomnia	18 (11.5%)
Headache	6 (3.9%)
Somnolence	5 (3.2%)
Irritability	5 (3.2%)
Anger	4 (2.6%)
Weight gain	4 (2.6%)
Fatigue	6 (3.9%)

Values are expressed as *N* (%). TEAEs, Treatment-emergent adverse events; SAS, safety analysis set.

Two (1.3%) patients experienced severe treatment-related TEAEs. A 12-year-old boy with a history of infantile purulent meningitis who was on concomitant sodium valproate developed aggression after 1 month of 6 mg perampanel add-on. He made full recovery after perampanel dose reduction. Another 21-year-old female patient with congenital cerebral dysplasia who was on concomitant levetiracetam attempted suicide (took 20 tablets of levetiracetam totaling 5,000 mg) after receiving 10 mg/day perampanel add-on for 20 days. She stopped using perampanel after gradual perampanel dose reduction.

Subgroup analyses found no significant differences in the incidences of treatment-related TEAEs and discontinuation due to TEAEs among patients taking concomitant sodium valproate, patients taking concomitant levetiracetam and those taking concomitant oxcarbazepine (Both *p* > 0.05; Figure 5). Additionally, there were no significant differences in the incidences of treatment-related TEAEs and discontinuation due to TEAEs between patients 12–18 years old and those >18 years old and between patients receiving perampanel as first add-on and those receiving perampanel as second add-on (all *p* > 0.05; Figures 4A,B).

## 4. Discussion

In this multicenter, single-arm, open label, phase 4 prospective clinical trial on efficacy and safety of perampanel early add-on treatment for FOS with or without FBTCS in Chinese patients aged ≥ 12 years, we found that at a mean maintenance perampanel dose of 5.9 ± 1.5 mg/day, our patients had a 72% 8-month retention rate. The 50% responder rate and seizure-freedom rate were 67.9% and 30.5%, respectively during maintenance. Among these patients, patients with FBTCS had higher 50% responder rate (96.0%) and seizure-freedom rate (76.0%). Additionally, 55.1% of our patients experienced treatment-related TEAEs, and the most common TEAEs were dizziness, hypersomnia, headache, somnolence and irritability. Withdrawal due to TEAEs occurred to 14.7% of the patients. Finally, whether patients were 12–18 years old or >18 years did not seem to affect the efficacy and safety of perampanel early add-on in our study, nor did whether perampanel was first add-on or second add-on. Patients’ concomitant ASMs did not affect the safety of the treatment although patients on concomitant sodium valproate had a significantly higher seizure-freedom rate than those on concomitant oxcarbazepine.

To the best of our knowledge, this is the first interventional study conducted in China on the efficacy and safety of perampanel in treating Chinese patients with FOS and the first study on perampanel early add-on conduct in China. Patients in our study had a 72% 8-month retention rate at a mean maintenance perampanel dose of 5.9 ± 1.5 mg/day. Our finding was consistent with previous studies (11, 18, 35, 36). A prospective observational study on perampanel early add-on in treating patients with FOS conducted in Spain reported a 12-month retention rate of 80.5% at a medium maintenance dose of 6 mg/day (35), and a 2-year real-world experience of perampanel add-on being used to treat patients with refractory epilepsy reported a 12-month retention rate of 55% at a medium maintenance dose of 6 mg/day (36). Our findings were also consistent with 2 previous observational studies on perampanel add-on conducted in China that reported 6-month retention rates of 77.8% and 67.9% at a mean maintenance dose of 4.96 ± 2.41 mg/day and 5.1 ± 1.5 mg/day, respectively (11, 18). The fact that the mean maintenance dose in our study was somewhat higher than these 2 observational studies could be attributed to the fact that as long as patients in our study could tolerate it, perampanel was up-titrated to 8 mg/day during titration regardless of whether their seizures could be controlled at lower dosage.

Patients in our study had a 67.9 50% responder rate and a 30.5% seizure-freedom rate during the 6-month maintenance, these results were in line with previous studies on perampanel early add-on (35, 37–39). In addition, consistent with previous studies reporting higher efficacy of perampanel add-on in patients with FBTCS compared to those without FBTCS (14, 19, 38, 40), we also found that perampanel was especially effective in patients with FBTCS, with a 50% responder rate of 96.0% and seizure-freedom rate of 76.0% during maintenance. It has been reported that AMPA receptors were involved in several disorders characterized by neuronal overexcitation and that generalized seizures were accompanied by abnormal cortical hyperexcitability that could be treated with ASM (41). In the rat amygdala-kindling model (a chronic model of partial seizures with secondary generalized seizures), perampanel increased the threshold of afterdischarge, shortened durations of motor seizures and afterdischarge, and reduced seizure severity, therefore, perampanel could inhibit both FOS and FBTCS (42, 43). It has also be observed that in some perampanel-treated animals, after complete inhibition of behavioral seizures, there was still afterdischarge, suggesting that perampanel might be more effective in inhibiting seizure propagation than initiation (43), this could explain better efficacy of perampanel in treating patients with FBTCS.

As observed by previous studies (35, 44–46), we also found a significantly higher seizure-freedom rate in patients on concomitant sodium valproate than those on concomitant oxcarbazepine. Since perampanel is primarily metabolized by cytochrome P450 (CYP) 3A4 in the liver, its clearance is increased by other CYP3A4 inducers such as oxcarbazepine and inhibited by CYP3A4 enzyme inhibiting ASMs such as sodium valproate (3, 35, 44, 45). Therefore, patients on concomitant enzyme inhibiting ASM(s) would have a higher plasma concentration of perampanel while patients on concomitant enzyme inducing ASM(s) could have a lower plasma concentration (3, 44). Our finding of better efficacy associated with perampanel + enzyme inhibiting sodium valproate than perampanel + enzyme inducing oxcarbazepine was consistent with clinical pharmacology of perampanel (3, 44). Further researches are needed to explore how to adjust dose of perampanel in patients taking concomitant enzyme inducing or inhibiting ASM(s) in order to achieve better seizure control.

Patients 12–18 years old and those >18 years old in our study had comparable 50% responder rates and seizure-freedom rates. Studies have not reached a consensus as to whether and/or how a patient’s age affected his/her response. Some studies found that chance of seizure-freedom increased with increasing age and older patients (>65 years old) responded better to perampanel (45–47), while other found that age did not seem to affect its efficacy (11, 18, 48). Difference in patient age ranges and methods of determining age-efficacy relationship adopted by difference studies could contribute to different conclusions reached by these studies.

In our study, whether perampanel was first add-on or second add-on in a patient did not affect its efficacy. A prospective observational study conducted in Spain found significantly higher seizure-freedom rate associated with perampanel first-add than second add-on (35), and several other studies reported that likelihood of seizure freedom in a patient receiving perampanel decreased with increasing number of previous ASM regimens (45, 46). As there were only 13 patients who took perampanel as second add-on in our study, whether perampanel as first add-on or second add-on in a patient affected its efficacy could not be properly determined.

Consistent with previous studies (16, 36, 45), 55.1% of our patients experienced treatment-related TEAEs, and the most common TEAEs were dizziness, hypersomnia, headache, somnolence and irritability. Withdrawal due to TEAEs occurred to 14.7% of the patients in our study. As perampanel has a long half-life (approximately 105 h), patients are advised to take it once daily at bedtime to alleviate the feelings of somnolence and dizziness (3). For patients who experience TEAEs during titration, slower titration is recommended, as it has been found that slow titration (2 mg every 3–4 weeks) could reduce the incidence of TEAEs including the incidence of psychiatric TEAEs (3, 10). For patients experiencing TEAEs during maintenance, a temporary dose reduction could be tried until the TEAEs resolve and the dose of perampanel could be up-titrated again when the patients become more tolerant to the treatment (10).

Two patients in our study experience severe treatment-related psychiatric TEAEs, one developed aggressive behavior and the other attempted suicide. Aggression and suicide attempt are both known TEAEs of perampanel treatment (3, 17, 40, 41). Patients with history of psychiatric comorbidities were at higher risk of developing psychiatric AEs, extra caution should be exercised when treating such patients. Patients’ mental state should be monitored especially in those with a history of psychiatric comorbidities (3).

Our study demonstrates that perampanel early add-on treatment was effective and safe in treating Chinese patients with FOS who failed ASM monotherapies. During recent years, some have argued for early use of combination therapy especially in patients with severe epilepsy who could tolerate their first ASM and were partially responsive (6, 7). Kwan et al. reported in 2000 that among 248 patients who had unsuccessful initial monotherapy, 116 patients received alternative monotherapy and had a seizure-freedom rate of 37%. However, among these 116 patients, 31 patients failed their initial monotherapy due to lack of efficacy and their seizure-freedom rate on alternative monotherapy was only 16% (49). In addition, for 56 patients whose seizures were inadequately controlled on their first tolerated ASM, the seizure-freedom rates in patients receiving add-on therapy and alternative monotherapy were 26 and 17%, respectively (49). On the other hand, none of the 11 patients receiving later add-on therapy after failed alternative monotherapy achieved seizure freedom (49). These findings suggests that patients who failed their first ASM due to lack of efficacy could benefits more from early combination immediately after their failed first monotherapy than later combination after failure of 2 monotherapy regimens (49). Chi et al. reported that patients receiving combination therapy after failure on their first ASM at >50% define daily dose were more likely to achieve seizure-freedom (59.8%) than those receiving alternative ASM monotherapy (28.9%) or initial ASM at increased dosage (16.5%) (8). Over the last 30 years, numerous newer ASMs with wide range of mechanisms of action have been introduced (50). The emergence of these new ASMs with wide range of mechanisms of action could make combination treatment more effective (9). A retrospective study in 2014 found that a higher percentage of patient with FOS achieved seizure freedom on dual therapy (38%) than patients in a similar study from the same center 10 years before (27%), and 8 newer ASMs unavailable in the previous study were used by patients in the 2014 study, suggesting that some patients with FOS could become seizure free with helps from the newer ASMs as add-on therapies (9). Furthermore, combining 2 ASMs targeting distinct pharmacological pathways could be more effective than combining 2 ASMs targeting the same pathway (50). Most patients who achieved seizure control did so with their first or second ASM regimen (10). In reality, many neurologists in China prefer combination therapy when a patient fails to respond to the initial ASM monotherapy (8). Our study set strict inclusion and exclusion criteria and added to and confirmed findings of previous studies that perampanel was effective and safe in treating patients with FOS (10–12, 14–18). Our study is especially meaningful for clinicians in China, as studies on perampanel in treating Chinese adult patients with epilepsy have been lacking (11, 14, 18, 19). Findings of our study could potentially help neurologist in China to better utilize perampanel in treating Chinese patients with FOS, especially those who failed 1 or 2 ASM monotherapies.

We did not assess whether perampanel early add-on affected patients’ cognitive functions in our study. ASMs could negatively affect patients’ cognitive functions, especially in children and adolescents as their brains are still developing (22, 23), and safety data for pediatric patients cannot be extrapolated from adult (24), it is therefore important to evaluate safety of adjunctive perampanel in pediatric patients. One observational study reported that adjunctive perampanel did not affect executive functions in adolescents with resistant FOS during a 12-month treatment period, and executive functions in several patients actually improved (25). Another observational study found that perampanel first add-on did not adversely affect the patients’ “non-verbal intelligence, executive functions, emotional/behavioral symptoms of children and parental stress levels” (26). A randomized, double-blind study on adolescent patients with FOS evaluated the effect of perampanel add-on on cognitive functions using Cognitive Drug Research (CDR) System Global Cognition Score and found that patients on perampanel add-on and those placebo had comparable global cognitive scores at the end of study (6-week titration +13-week maintenance), although patients on perampanel add-on was worse in 2 subdomain and better in 1 subdomain than patients receiving placebo (51). A systematic review done by Witt et al. suggested that perampanel treatment had an overall neutral cognitive profile with “no systematic cognitive deteriorations or improvements” (52). Whether perampanel early add-on has detrimental effect on cognitive functions of Chinese patients with FOS needs to be explored in future studies.

Our study has several limitations. First, the maintenance period in our study was 24 weeks, and as such, long-term efficacy and safety of perampanel early add-on could not be assessed by our study. Secondly, some of the subgroups used in our subgroup analysis had relatively small sample size such as the subgroup of patients receiving perampanel as second add-on that included 13 patients, a small sample size might affect results of the subgroup analysis. Thirdly, as etiologies of epilepsy, age of seizure onset and whether the included patients have genetic syndromes or associated neurodevelopmental disorders were not collected for every patient in our study, how these factors might affect efficacy and safety of perampanel could not be assess by our study. Finally, like some previous studies on ASMs that included adult patients and a small number of pediatric patients, as observed by the systematic review done by Rosati et al. (53), our study also included much more patients ≥18 years old than patients <18 years old (26). Lack of funding and ethical consideration played a role. It is possible that such imbalance in patient numbers might affect efficacy and safety comparison between these 2 groups of patients (Figure 4). However, despite these limitations, our study included 150 patients from multiple provinces in China, and it is the first clinical trial on efficacy and safety of perampanel in treating patients with epilepsy in China, and as such, it could provide useful information to epilepsy specialists in China and expand the treatment options for Chinese patients with FOS.

In conclusion, perampanel early add-on therapy is effective and safe in treating Chinese patients≥12 years old with FOS with or without FBTCS, and patients with FBTCS have better clinical response to the treatment. Dose adjustment should be considered in patients taking concomitant enzyme inducing or inhibiting ASMs.

## Data availability statement

The raw data supporting the conclusions of this article will be made available by the authors, without undue reservation.

## Ethics statement

This study was reviewed and approved by the Institutional Review Boards for Ethical Approval of all participating hospitals in 2020. The participating hospitals were as follows: (1) Xuanwu Hospital, Capital Medical University, (2) Peking Union Medical College Hospital, (3) The First Hospital Of Jilin University, (4) Tianjin Huanhu Hospital, (5) The First Affiliated Hospital of USTC, Division of Life Sciences and Medicine, University of Science and Technology of China, (6) Beijing Tiantan Hospital, Capital Medical University, (7) Shenzhen People’s Hospital, The Second Clinical Medical College, Jinan University, The First Affiliated Hospital, Southern University of Science and Technology, (8) The Second Affiliated Hospital of Guangzhou Medical University, Key Laboratory of Neurogenetics and Channelopathies of Guangdong Province and the Ministry of Education of China, (9) Huashan Hospital, Fudan University, (10) Peking University First Hospital, (11) Xiangya Hospital, Central South University, (12) The Affiliated Hospital of Xuzhou Medical University, (13) Children’s Hospital, Zhejiang University School of Medicine, (14) Wuhan Children’s Hospital, Tongji Medical College, Huazhong University of Science and Technology, and (15) Tianjin Union Medical Center. The study was conducted in accordance with the principles of the Declaration of Helsinki. All patients or their legal guardian/next of kin gave written informed consent to participate in this study before enrollment. This trial was registered at Chinese Clinical Trial Registry (www.chictr.org.cn, Identifier: ChiCTR2000039510).

## Author contributions

LG and YW were in charge of the conception of the study and data analysis and interpretation. LG, QL, ZW, WY, GW, XS, YG, YY, ZH, YJ, BX, GC, FG, JH, JL, MZ, and YW contributed to study design and data acquisition, revised it critically for important intellectual content, and provided approval for publication of the content and agree to be accountable for all aspects of the work in ensuring that questions related to the accuracy or integrity of any part of the work are appropriately investigated and resolved. GL wrote the first draft of the manuscript. All authors contributed to the article and approved the submitted version.

## Funding

This study was funded by Eisai (China) Pharmaceutical Co., Ltd. and China Association against Epilepsy. The funders had no role in the study design, data collection and analysis, decision to publish, or manuscript preparation.

## Conflict of interest

The authors declare that the research was conducted in the absence of any commercial or financial relationships that could be construed as a potential conflict of interest.

## Publisher’s note

All claims expressed in this article are solely those of the authors and do not necessarily represent those of their affiliated organizations, or those of the publisher, the editors and the reviewers. Any product that may be evaluated in this article, or claim that may be made by its manufacturer, is not guaranteed or endorsed by the publisher.
